# Effect of Dipeptidyl-Peptidase 4 Inhibitors on Circulating Oxidative Stress Biomarkers in Patients with Type 2 Diabetes Mellitus

**DOI:** 10.3390/antiox9030233

**Published:** 2020-03-11

**Authors:** Elisabetta Bigagli, Cristina Luceri, Ilaria Dicembrini, Lorenzo Tatti, Francesca Scavone, Lisa Giovannelli, Edoardo Mannucci, Maura Lodovici

**Affiliations:** 1 Department of Neurosciences, Psychology, Drug Research and Child Health (NEUROFARBA), Section of Pharmacology and Toxicology, University of Florence, 50139 Florence, Italy; lorenzo.tatti.95@gmail.com (L.T.); francesca.scavone@unifi.it (F.S.); lisa.giovannelli@unifi.it (L.G.); maura.lodovici@unifi.it (M.L.); 2 Department of Clinical and Experimental Biomedical Sciences, University of Florence and Diabetology Unit, Careggi Hospital, 50139 Florence, Italy; ilaria.dicembrini@unifi.it (I.D.); edoardo.mannucci@unifi.it (E.M.)

**Keywords:** type 2 diabetes, dipeptidyl peptidase-4 inhibitors, biomarkers, oxidative stress

## Abstract

Pre-clinical studies suggested potential cardiovascular benefits of dipeptidyl peptidase-4 inhibitors (DPP4i), however, clinical trials showed neither beneficial nor detrimental effects in patients with type 2 diabetes mellitus (T2DM). We examined the effects of DPP4i on several circulating oxidative stress markers in a cohort of 32 T2DM patients (21 males and 11 post-menopausal females), who were already on routine antidiabetic treatment. Propensity score matching was used to adjust demographic and clinical characteristics between patients who received and who did not receive DPP4i. Whole-blood reactive oxygen species (ROS), plasma advanced glycation end products (AGEs), advanced oxidation protein products (AOPP), carbonyl residues, as well as ferric reducing ability of plasma (FRAP) and leukocyte DNA oxidative damage (Fpg sites), were evaluated. With the exception of Fpg sites, that showed a borderline increase in DPP4i users compared to non-users (*p* = 0.0507), none of the biomarkers measured was affected by DPP4i treatment. An inverse correlation between estimated glomerular filtration rate and AGEs (*p* < 0.0001) and Fpg sites (*p* < 0.05) was also observed. This study does not show any major effect of DPP4i on oxidative stress, assessed by several circulating biomarkers of oxidative damage, in propensity score-matched cohorts of T2DM patients.

## 1. Introduction

Dipeptidyl peptidase-4 inhibitors (DPP4i) are oral agents used for the pharmacological treatment of adults with type 2 diabetes mellitus (T2DM). By preventing glucagon-like peptide 1 (GLP-1) breakdown, DPP4i enhance endogenous insulin secretion and suppress that of glucagon, resulting in the reduction of blood glucose levels [1]. Several clinical trials demonstrated that these agents are effective in reducing glycated hemoglobin (HbA1c) with a low risk of hypoglycemia and neutral effects on weight, compared to sulphonylureas [2,3,4].

A meta-analysis of short and medium-term trials with metabolic endpoints, showed that the treatment with DPP4i was associated with reduced incidence of major adverse cardiovascular events (MACE) and all-cause mortality [5]. Later on, trials with cardiovascular events as major endpoints, showed a neutral effect of DPP4i with respect to the incidence of MACE in T2DM patients with cardiovascular disease [6,7,8]. Moreover, a post hoc analysis from the SAVOR-TIMI 53, found a moderate increase in the risk of hospitalization due to hearth failure with saxagliptin compared to placebo [6], not observed in the EXAMINE and TECOS trials with alogliptin and sitagliptin [8,9]. More recently, in the CARMELINA trial, no increased risk of heart failure with linagliptin compared to placebo was reported in patients at high cardio renal risk [10]. The results of the CAROLINA trial also demonstrated that linagliptin was non-inferior to glimepiride on the prevention of MACE in patients with elevated cardiovascular risk [11].

Oxidative stress is a significant risk factor in the development and progression of T2DM and associated vascular complications [12,13,14]. Increased levels of circulating oxidative stress biomarkers have been consistently found in T2DM patients compared to healthy controls as well in T2DM patients with micro and macrovascular complications compared to those without [15].

Experimental in vitro and in vivo studies showed that DPP4i protect against oxidative stress, suggesting potential cardiovascular benefits that have not been observed in human studies. In endothelial and tubular cells, linagliptin inhibited reactive oxygen species (ROS) production and inflammation induced by advanced glycation end products (AGEs) [16,17]. Vildagliptin attenuated vascular injury by suppressing the AGEs-receptor for AGEs (RAGE)-oxidative stress axis in diabetic rats [18]. Saxagliptin prevented vascular remodeling and oxidative stress in T2DM mice by abolishing nicotinamide adenine dinucleotide phosphate NAD(P)H oxidase-driven endothelial nitric oxide synthase (eNOS) uncoupling [19] and prevented increased coronary artery stiffness and collagen deposition in a mini swine model of heart failure, partly by decreasing AGEs [20]. However, prolonged DPP4 inhibition let to the development of cardiac fibrosis in old, diabetic, high fat-fed mice [21].

Some studies were also conducted in T2DM patients, yielding conflicting results: isoprostanes, markers of lipid peroxidation, were not affected by the addition of sitagliptin to metformin [22]; similarly, no effect of vildagliptin on urinary isoprostanes levels [23] and of alogliptin on circulating AGEs, was observed [24]. Nomoto et al. demonstrated that sitagliptin improved antioxidant capacity but did not modify reactive oxygen metabolites-derived compounds (d-ROMs) levels [25]. However, some positive results were also reported: serum malondialdehyde-modified low-density lipoproteins and the soluble form of RAGE were reduced after treatment with linagliptin [26] and alogliptin [24], and both sitagliptin and vildagliptin reduced plasma nitrotyrosine levels [27].

These controversial results led us to further explore the effects of DPP4i on oxidative stress in patients with T2DM who were already on other antidiabetic treatment, evaluating a set of circulating biomarkers of oxidative damage to lipids, proteins and DNA.

## 2. Methods

### 2.1. Patients

Thirty-two T2DM patients (21 males and 11 post-menopausal females) from the Diabetology Unit, Careggi Teaching Hospital, Florence, Italy, were included in this study. Patients were given detailed explanations of the study protocol and all patients provided written informed consent prior to enrollment. The study protocol was approved by the Ethical Committee of Careggi Teaching Hospital, Florence, Italy on October, 25, 2018, registry number 13469_bio.

The inclusion criteria were as follows: (1) diagnosis of T2DM, (2) age ≥ 40 years, (3) treatment with diet and/or metformin and/or basal insulin analogues and/or DPP-4i and (4) no modifications of the antidiabetic therapy within the last 6 months.

The exclusion criteria were as follows: (1) use of antioxidant supplements (2) treatment with SGLT-2 inhibitors (3) treatment with GLP-1 receptor agonists.

Demographic and clinical characteristics obtained from the medical record included age, sex, height, weight, body mass index (BMI) and lipid profiles. The patients’ history of coronary heart disease, peripheral vascular disease, diabetic retinopathy and nephropathy were also collected from the hospital records. Peripheral blood samples were collected by venipuncture into ethylenediaminetetraacetic acid (EDTA)-treated tubes and all plasma samples were stored at −20 °C until analysis, which was undertaken within 30 days.

### 2.2. Reactive Oxygen Species (ROS) Determination

ROS were determined using the Free Oxygen Radical Testing (Callegari 1930, Parma, Italy) according to [28]. Whole blood (20 µL) was added to acidic buffer and to phenylenediamine derivative [2CrNH2]. Samples were then centrifuged (3500 rpm) for 1 min and incubated for 6 min at room temperature. Absorbance was determined at 505 nm.

### 2.3. Ferric Reducing Ability of Plasma (FRAP)

The ferric reducing ability of plasma (FRAP) assay was performed to measure the total antioxidant capacity of plasma, according to the method by Benzie and Strain [29]. Plasma samples (30 μL) were added to 90 μL of distilled water and freshly prepared FRAP solution (300 mM acetate buffer (pH 3.6), 10 mM 2,4,6-tripyridyl-*S*-triazine (TPTZ) in 40 mM HCl and 20 mM FeCl3·6H2O (10:1:1)). The absorbance was measured at 595 nm. FRAP was estimated from a standard curve of FeSO_4_·7H_2_O and expressed in µM. All the reagents were purchased by Sigma Aldrich, Milan, Italy.

### 2.4. Advanced Oxidation Protein Product (AOPP)

Advanced oxidation protein product (AOPP) levels, markers of protein oxidation and inflammation, were determined by using 20 µL of plasma added to 980 µL of potassium phosphate buffer (PBS), 50 µL of KI 1.16 M and 100 µL of acetic acid according to the method by Witko-Sarsat et al. [30]. The absorbance was read at 340 nm. AOPP were expressed as µmol/mg of proteins. Chloramine-T (Sigma-Aldrich, Milan, Italy) was used for the calibration curve.

### 2.5. Carbonyl Residues

Carbonyl residues, markers of protein oxidation, were determined using the method of Correa-Salde and Albesa [31]. Plasma samples (100 µL) were derivatized with 900 µL of 0.1% dinitrophenylhydrazine in 2 M HCl (Sigma Aldrich, Milan, Italy city, country), for 1 h at room temperature (RT) followed by the addition of 400 µL of 10% trichloroacetic acid (TCA) and centrifugation at 10,000× *g* for 20 min at 4 °C. The pellets were washed three times with ethanol/ethyl acetate (1:1) and centrifuged at 10,000× *g* for 3 min at 4 °C. The pellets were then dissolved in 1.5 mL guanidine HCl (6M) in 20 mM phosphate-buffered saline (PBS) pH 7.5 and incubated at 37 °C for 30 min. Insoluble debris were removed by centrifugation. Carbonyl residues were calculated from absorbance readings at 370 nm using a molar absorption coefficient of 22,000 M^−1^ cm^−1^ and expressed as nmol/g of proteins. Protein content was measured with the Bio-Rad DC protein assay kit (Bio-Rad, Milan, Italy).

### 2.6. Thiobarbituric Acid Reactive Substances (TBARS)

Thiobarbituric acid reactive substances (TBARS), markers of lipid peroxidation, were evaluated using 100 µL of plasma, according to the method by [32]. Briefly, after the addition of 100 µL TCA, the resulting supernatant (160 µL) was added to 32 µL thiobarbituric acid, 0.12 M (Sigma-Aldrich, Milan, Italy city, country) and heated at 100 °C, for 15 min. The samples were then placed for 10 min in ice and centrifuged at 1600× *g* at 4 °C, for 10 min. The absorbance of the supernatants was measured at 532 nm by using a Wallac 1420 Victor3 Multilabel Counter (Perkin Elmer, Waltham, MA, USAmanufacturer, city, country). The amount of TBARS, expressed as µM, was calculated using a molar absorption coefficient of 1.56 × 10^−5^ M^−1^ cm^−1^.

### 2.7. Advanced Glycated End-Products (AGEs)

Plasma samples (100 µL) were diluted in H_2_O (1:5) and fluorescence intensity was read at 460 nm, after excitation at 355 nm. AGEs levels were expressed in arbitrary units (AU) [33].

### 2.8. FPG Sites

For the analysis of DNA damage in peripheral blood cells, whole blood aliquots (100 µL) were frozen at −80 °C as described in [34] and stored for 3 months before analysis. Briefly, the comet assay was performed as follows: 50 μL of cell suspension containing 10 μL of frozen whole blood and 40 μL Roswell Park Memorial Institute (RPMI) were mixed with 150 μL 1% low melting-point agarose in PBS in order to achieve 0.75% LMP agarose final concentration, and used to prepare duplicate gels for each patient (20,000 cells/gel) on Gelbond Films (Lonza, Basel, Switzerland). As a reference control for inter-experimental variability of electrophoresis conditions, we used HeLa cells aliquots prepared from a single culture and stored at −80 °C. One aliquot was used in each experiment to prepare 2 gels (20,000 cells/gel) and run with the experimental samples. We then followed the procedure described in [34]. For oxidatively generated damage detection, the enzyme formamidopyrimidine DNA glycosylase (Fpg) enzyme was used (crude E. Coli extract kindly provided by Prof. A.R. Collins, University of Oslo, Norway) at 1:1000 dilution, i.e., the optimum concentration established by titration.

### 2.9. Statistical Analyses

The Kolmogorov–Smirnov test was used to verify the normal distribution of the results. Continuous variables and those that resulted to be normally distributed were expressed as means ± standard deviation (SD). When data were not normally distributed, they were reported as median and interquartile range.

For the analysis, we used propensity score matching to adjust patient characteristics and disease severity between the two groups.

Among demographic and clinical characteristics at baseline of enrolled patients, putative determinants of referral to the DPP-4i treatment were identified in the whole sample by means of comparisons between treated and untreated subjects, using chi square tests for categorical variables and either unpaired Student’s *t* test or Mann–Whitney test depending on the (normal or non-normal) distribution.

Variables significantly associated to the DPP-4i treatment were then inputted as covariates in a logistic regression model, with DPP-4i treatment (0/1) as dependent variable. The model was used to build an equation for the calculation of a propensity score. We estimated the propensity score using a logistic regression model, in which individuals at the time of hospital admission was regressed on measured baseline characteristics. Covariates for inclusion in the propensity score model were chosen based on their potential to be associated with the outcomes. Propensity score-matched analyses, using the 1:1 nearest neighbor technique with a small caliper of 0.05, were carried out to ensure better balance.

The propensity score-matched two cohorts were therefore compared for DPP-4i treatment in order to evaluate differences in the levels of a set of circulating biomarkers of oxidative damage to lipids, proteins and DNA.

Comparison of continuous variables between matched pairs were performed using the paired *t*-test (when data were normally distributed) or Wilcoxon test (when data were non-normally distributed). Differences between proportions were assessed using the Fisher exact test. *p* values < 0.05 were considered significant.

## 3. Results

### 3.1. Clinical Characteristics of the Patients

The clinical characteristics of the study groups after propensity score matching are shown in Table 1. After propensity score matching, there were no significant differences between the two groups in the levels of HbA1c and lipid profiles, body mass index (BMI) and in the male-to-female ratio. The distribution of diabetes-related micro and macro vascular complications was also similar. However, duration of diabetes was significantly higher in the DPP4i group compared to those who did not receive the treatment (*p* < 0.01). eGFR was also significantly reduced in T2DM patients treated with DPP4i compared to those not treated (*p* < 0.01).

### 3.2. Plasma Oxidative Stress Markers, Antioxidant Status and Oxidative DNA Damage

**Figure 1:**
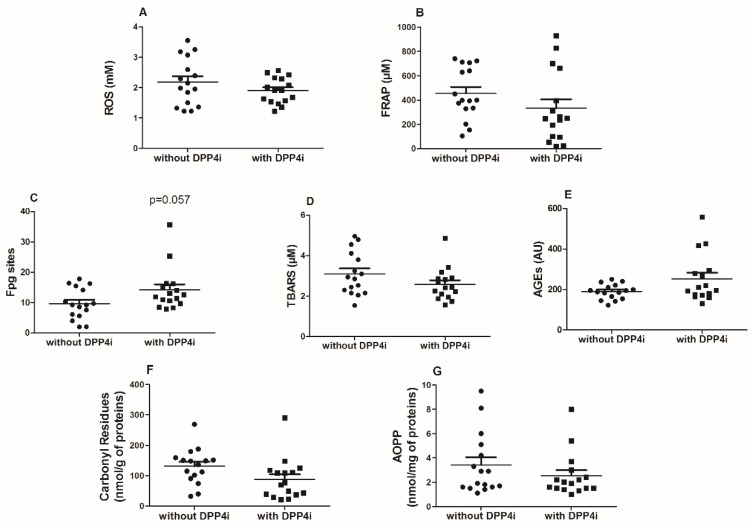
Scatter dot plot of reactive oxygen species (ROS) (panel **A**), ferric reducing ability of plasma (FRAP) (panel **B**) Fpg sites (panel **C**) thiobarbituric acid reactive substances (TBARS, panel **D**), advanced glycation end products (AGEs) (panel **E**), carbonyl residues (panel **F**), and advanced oxidation protein products (AOPP) levels (panel **G**) in type 2 diabetes mellitus (T2DM) patients with and without treatment with dipeptidyl peptidase-4 inhibitors (DPP4i). Comparison of continuous variables between matched pairs were performed using the paired *t*-test (when data were normally distributed) or Wilcoxon test (when data were non-normally distributed).

After propensity score matching, the plasma levels of AGEs, ROS, TBARS, AOPP and carbonyl residues were not significantly different in T2DM patients taking DPP4i compared with non-users. The plasma antioxidant capacity (FRAP) between patients taking DPP4i was also similar to those not treated (Figure 1, panels A,B,D,E,F,G). On the contrary, Fpg sites were borderline significantly higher in DPP4i users in comparison with matched non-users (*p* = 0.0507) (Figure 1, panel C). Several positive correlations among oxidative stress biomarkers were found; in particular, a strong correlation between TBARS and AOPP (*p* < 0.001) and between carbonyl residues and AOPP (*p* < 0.0001) were observed. Interestingly, Fpg sites and AGEs, were inversely associated with eGFR (*p* < 0.05 and *p* < 0.0001, respectively). Fpg sites were also correlated with the duration of diabetes and AGEs with the age of the patients (Table 2).

## 4. Discussion

The present data do not suggest any major effect of DPP4i on oxidative stress, as explored through the measurement of several circulating biomarkers. This result is at variance with those of several experimental studies, in vitro and in vivo on animal models of diabetes, showing a protective effect of DPP4i [16,17,18,19,20]. Differences from animal studies could be attributed either to diversities across species or to differences in circulating drug concentrations.

Some previous studies explored the effects of DPP4i on oxidative stress markers. In randomized, active-comparator-controlled trials, sitagliptin and vildagliptin failed to produce any effect on lipid peroxidation markers, in line with our results on TBARS [22,23]. Consistent with our findings, circulating levels of AGEs were not modified by alogliptin [24] and sitagliptin did not reduce ROS levels [25]. Conversely, a single-arm trial with linagliptin, revealed a beneficial effect of the treatment, when compared to baseline, on malondialdehyde-modified LDL; however, there was a concomitant reduction of mean glucose and HbA1c, which could explain the improvement of oxidative status [26]. Similarly, in the study by Rizzo and collaborators, the beneficial effects of sitagliptin and vildagliptin on nitrotyrosine plasma levels were associated to a significant decline in HbA1c and glucose levels and to a better control of daily glucose excursions [27]. Sakata et al. [24] demonstrated that alogliptin reduced AGE levels only in patients with higher values of AGEs at baseline and this decrease was positively correlated with that of HbA1c; however, when all patients were analyzed, no significant effect was observed.

In our study, the difference in glycemic control between cases and controls was very small, since both groups were extracted from a cohort receiving accurate treatment for T2DM. Therefore, the present study could underestimate the global effect of treatment with DPP4i, since the possible benefits of blood glucose reduction are blunted because of study design. The enrolled cohorts showed, on average, a satisfactory glycemic control, with a low dispersion of HbA1c values. This prevents any analysis on possible effects of hyperglycemia on oxidative stress. It is also important to highlight that the levels of oxidative stress biomarkers in the entire cohort of T2DM patients did not significantly differ from those measured in the serum of healthy controls [35]. We demonstrated previously that significant differences in FRAP levels were seen between heathy controls and poorly controlled T2DM patients but not between heathy controls and T2DM patients with good glycemic control [36]; similarly, Morsi et al. [37] did not find significant differences in malondialdehyde (MDA) levels between controls and well controlled T2DM patients. However, in heathy volunteers, Fpg sites, marker of oxidative damage, were reduced compared to well controlled T2DM patients [36]. Indeed, Fpg sites levels were the sole biomarker showing a borderline difference between patients taking DPP4i and those not treated.

Another limitation of this study, is represented by the fact that propensity score matching did not prevent a significant difference in estimated glomerular filtration rate between cases and controls. Renal failure is associated with oxidative stress [38]; this is consistent with the inverse correlation of estimated glomerular filtration rate with AGEs and Fpg sites observed in the present study. As a consequence, there could be a bias in favor of controls with respect to biomarkers of oxidative stress. It is also worth to highlight that although intriguing, the borderline increase in the levels of Fpg sites observed in patients taking DPP4i, is strongly linked to the presence of a single patient with particularly high levels of Fpg sites.

On the other hand, some strengths of this study should also be recognized. Propensity score matching warrants a greater reliability of results than traditional observational data. In addition, the selection of patients from a larger clinical cohort accounts for a greater representativeness of samples in comparison with randomized trials. Study procedures on patients were kept to a minimum (only one venipuncture with a blood sample), in order to remain close to routine clinical practice. A further strength is represented by the fact that several biomarkers of oxidative damage were assessed, with concordant results across different markers. Conversely, studies focused on the evaluation of a single oxidative damage marker may give incomplete information on the overall redox status. Notably, we measured various biomarkers reflecting oxidative damage to the main macromolecules such as lipids (TBARS), proteins (carbonyl residues and AOPPs) and DNA (Fpg sites) and found positive correlations among almost all of them, in line with our previous results [35].

All these biomarkers share some advantages including relatively easy detection and possibility to be used in large scale; carbonyl residues are highly stable products and AOPP are also considered inflammatory markers. However, some limitations should be also accounted: TBARS has been employed for MDA determination but thiobarbituric acid TBA may also react with other aldehydes; the FRAP assay is reliable for detecting uric acid, α-tocopherol, ascorbic acid and bilirubin, but may underestimate albumin and glutathione GSH content [39]. Another important issue also highlighted in our previous work [15], is the lack of standardization among methods employed for determining oxidative stress biomarkers, the different biological fluids used, the absence of reference range intervals and validation in prospective studies.

However, several data suggest that oxidative damage is associated to cardiovascular diseases: increased carbonyl residues, AGEs and AOPPs were found in T2DM patients with cardiovascular complications compared to those without [40,41]. Increased AOPP plasma levels were considered a risk factor for endothelial dysfunction in T2DM patients without albuminuria [42]. AOPP levels were also higher in hypertensive patients and in particular, in those with renal complications [43]. In a prospective study, oxidative DNA damage, measured as 8-hydroxy-2-deoxyguanosine (8-OHdG), predicted micro and macrovascular complications in T2DM patients [44]. Experimental studies also highlighted the pathological role of some of these markers: by binding to their common receptor RAGE, AOPP and AGEs activate inflammatory and apoptotic pathways leading to oxidative stress, inflammation and cell death both on cardiomyocytes and renal cells [45,46,47].

The results of the present study are consistent with those of available cardiovascular outcome trials, which failed to show either beneficial or detrimental effects of DPP4i on major cardiovascular events [6,7,8]. Considering that oxidative stress is a well-established risk factor for atherogenesis and cardiovascular diseases, pharmacological strategies able to limit oxidative stress in humans should theoretically produce a reduction of cardiovascular risk. Results from the Cardiovascular and Renal Microvascular Outcome Study with Linagliptin in Patients with Type 2 Diabetes Mellitus (CARMELINA) study [10], performed in patients at high renal risk, suggested a protective effect of linagliptin on microalbuminuria; this result was not confirmed by other large-scale trials enrolling patients at lower risk of renal disease [48]. Oxidative stress is thought to play a role in the pathogenesis of microvascular complications of diabetes [12,49]; however, our results suggest that the hypothetical protective effect of DPP4i on microalbuminuria does not appear to be mediated by a reduction of substrate oxidation.

In conclusion, this observational study assessing several biomarkers of oxidative damage in propensity score-matched cohorts does not suggest any major effect of DPP4i on oxidative stress in humans.

## Figures and Tables

**Table 1:** Demographic and clinical characteristics of the study groups after propensity score matching.

	−DDP4i	+DDP4i	*p* Value
Number of Patients	16	16	
Gender F/M	5/11	6/10	ns
Age (yrs)	69.8 ± 2.2	72.38 ± 2.98	ns
Body Mass Index (BMI)	28.12 ± 1.33	25.6 ± 1.26	ns
Duration of Diabetes (yrs)	7.87 ± 1.81	20.94 ± 3.18	0.0015
HbA1c (mmol/mol)	52 (43.75–63.25)	49 (45.25–54)	ns
Total Cholesterol (mg/dL)	166.2 ± 10.14	152.2 ± 8.07	ns
High Density Lipoprotein (HDL) (mg/dL)	47.5 (37.5–56)	49.5 (41.75–56)	ns
Triglycerides (mg/dL)	144.9 ± 13.48	122.1 ± 11.14	ns
Estimated glomerular filtration rate (eGRF) (mL/min)	79 (68.5–90)	54.5 (27.25–89.75)	0.0027
Micro Complications (yes/no)	4/12	10/6	ns
Macro Complications (yes/no)	4/12	5/11	ns

Data are expressed as means ± standard deviation (SD). Non-normally distributed variables are expressed as median and interquartile range.

**Table 2:** Correlations between changes in oxidative stress-related biomarkers, duration of diabetes, eGRF and the age of the patients.

	Duration	eGRF	Age	AOPP	AGEs	TBARS	FRAP	ROS	Carbonyl Residues
Duration									
eGRF	−0.6934 ****								
Age	0.4243 *	−0.5970 ***							
AOPP	−0.2149	0.0421	0.2980						
AGEs	0.6587 ****	−0.6825 ****	0.5500 **	−0.1214					
TBARS	−0.3783 *	0.0814	0.1575	0.6358 ***	−0.2406				
FRAP	−0.0172	0.2041	0.0182	0.0411	0.0405	0.0592			
ROS	−0.1504	0.1554	−0.0942	−0.2483	−0.1995	−0.0253	−0.1191		
Carbonyl Residues	−0.2661	0.1154	0.2785	0.6948 ****	0.0514	0.5342 **	0.3504 *	−0.2148	
Fpg Sites	0.5659 ***	−0.4055 *	0.3473	−0.1654	0.3887 *	−0.2485	0.2160	0.0257	0.0206

**** *p* < 0.0001; *** *p* < 0.001; ** *p* < 0.01; * *p* < 0.05. eGRF: estimated glomerular filtration rate; AOPP: Advanced oxidation protein products; AGEs: advanced glycation end products; TBARS: thiobarbituric acid reactive substances; FRAP: ferric reducing ability of plasma; Fpg sites: formamidopyrimidine DNA glycosylase sites.
